# Public and Patient Involvement and Engagement in Clinical Trials: A Multi‐Perspective Mixed‐Methods Evaluation of the ROWTATE Programme

**DOI:** 10.1111/hex.70636

**Published:** 2026-03-12

**Authors:** Blerina Kellezi, Heather Peacock, Trevor Jones, Ali Gibson, Isabel Andrews, Steve Fallon, Rebecca Lindley, Kate Radford, Kay Bridger, Claire Mann, Cristina Roadevin, Marilyn James, Denise Kendrick

**Affiliations:** ^1^ Department of Psychology Nottingham Trent University Nottingham UK; ^2^ Centre for Academic Primary Care, School of Medicine University of Nottingham Nottingham UK; ^3^ Centre for Rehabilitation and Ageing Research, School of Medicine University of Nottingham Nottingham UK; ^4^ Nottingham Biomedical Research Centre Nottingham UK; ^5^ Nottingham Clinical Trials Unit, School of Medicine University of Nottingham Nottingham UK

**Keywords:** clinical trials, complex research programme, evaluation, mixed‐methods, PPIE

## Abstract

**Background:**

Patient and public involvement and engagement (PPIE) has many benefits for the design, delivery and dissemination of health research, but this can be difficult to achieve. Systematic reporting and evaluation of PPIE in multi‐year, multisite and complex clinical trials is very limited.

**Methodology:**

This evaluation presents a multi‐perspective description and evaluation of patient and public involvement within a large‐scale, multisite, longitudinal research programme focused on developing and evaluating a vocational rehabilitation and clinical psychology intervention for individuals recovering from traumatic injury. Conducted as part of the NIHR‐funded ROWTATE research programme (2019–2026), the evaluation explores the scope, impact, and lived experiences of PPIE group members across all phases of the study, from development of the intervention, feasibility study and trial design to intervention delivery, data collection, analysis and dissemination. Employing multiple methods, including open‐ended surveys with PPIE group members and researchers, a ‘You Said, We Did’ activity log, and minute taking, the study identifies key patterns related to PPIE group members' motivations, contributions, personal impact and barriers and facilitators to engagement.

**Results:**

Findings highlight the significant value of PPIE in enhancing study relevance, improving data collection and communication strategies, informing rehabilitation, clinical psychology and health economics components, and shaping intervention delivery. PPIE group members reported a strong sense of purpose and intellectual engagement, despite challenges including communication gaps and role clarity. Researchers valued PPIE contribution to the research, and the positive and enriching experience of working with PPIE group members. Both groups reflected on the barriers and facilitators to PPIE.

**Conclusion:**

The evaluation highlights the importance of inclusive, well‐supported, and transparent PPIE practices and contributes novel insights into the PPIE role in under‐researched domains such as clinical psychology and health economics.

**Patient or Public Contribution:**

Traumatic injury survivors were involved in all processes of this evaluation. This includes research design, funding acquisition, data collection, data analysis and interpretation and the write‐up of this manuscript. Five traumatic injury survivors are co‐authors of this manuscript.

## 1 Introduction

Working with people and communities, otherwise known as patient and public involvement and engagement (PPIE), is defined as a collaborative approach to research, wherein members of the public actively engage as partners in the research process, rather than solely as subjects of study [1]. PPIE can provide the unique perspective and knowledge of lived experiences [2], leading to research that is more directly related to the patients' concerns and needs [3]. PPIE contribution is also a democratic right where those most affected participate in health and social care research that helps address the impact of that particular issue [4].

There is strong evidence that PPIE in health services improves research quality for example, study design [5, 6, 7], outcomes [3] and impact [6, 8]. These benefits are crucial in clinical trial research, which is particularly difficult to develop and deliver [9]. Evidence from clinical trials has identified several benefits of PPIE in specific research processes including: making improvements to the research design such as adding a control group or developing interview topic guides [10, 11, 12, 13, 14], involvement in data collection such as interviews [10, 14], taking an active role in data analysis including data coding, theme generation [10, 14, 15], selecting appropriate outcomes [16] contributing to study outputs [13], and supporting the development of training for therapists and/or of study recruiters [13, 17].

While PPIE contributions in the design, execution and translation of research can be extensive [3], and routinely required by some funders (including being made mandatory by NIHR in the UK), a recent review shows that a large proportion of studies (80% of those not‐NIHR funded) do not include PPIE [18]. Those that do use PPIE in limited activities and provide no justification for doing so [19] or provide limited or inconsistent descriptions [20]. Although the quality of description is likely to improve with the development of guidelines for reporting PPIE, such as GRIPP2 [21], a detailed understanding and evaluation of PPIE in research, especially in clinical trials, remains limited.

Understanding and evaluation are important because meaningful PPIE is not easy to achieve or maintain over time [22]. Several studies acknowledge issues of power imbalances, costs and tokenism (e.g., [3, 23, 24, 25]), resources and infrastructure [21] which must be addressed for PPIE to be fully embedded as usual practice in research. This is especially important as PPIE can take significant resources, and several studies question whether the benefits justify the costs [24].

PPIE research also tends to overlook psychological theories that explain what makes engagement meaningful within specific contexts, such as the loss of social relationships, major life transitions, and trauma following illness or injury. For instance, in the context of traumatic injuries, PPIE group members often face profound life changes and a range of short‐ and long‐term social, psychological, physical, and economic consequences. These challenges can shape their ability to access and navigate care [26] and to communicate effectively [27]. Theoretical models such as The Social Identity Model of Trauma and Identity Change (SIMTIC [28]) help explain how important meaningful engagement can be undermined or achieved in times of transition after trauma where key identities (such as work or membership to sport groups) can be lost adding to the psychological distress. According to SIMTIC, acquisition of new and meaningful identities can be beneficial for health and wellbeing. PPIE offers an opportunity to build new identities which can help overcome the impact of loss, stigma and trauma [29].

Thus, the quality of PPIE experience and impact on PPIE group members themselves is very important. The UK Standards for Public Involvement [30, 31] state that excellent PPIE is inclusive, values all contributions, ensures people have a meaningful say in what happens and influences outcomes. While some trials have identified PPIE benefits, the extent and method of engagement across the stages of the research has not been described or evaluated in detail [5, 32, 33]. Moreover, it is not clear how to achieve and maintain this consistently over time while ensuring benefits of PPIE for the research and on PPIE group members themselves [34]. Finally, it is not clear how the experience of change, loss and trauma can impact this participation and satisfaction with the PPIE role. This paper addresses these research gaps.

### 1.1 The Present Evaluation

This paper takes a systematic approach to evaluating the experience and impact of PPIE in the development and delivery of the NIHR‐funded ROWTATE programme of research (2019–2026) which includes a clinical trial. ROWTATE develops and tests a vocational rehabilitation and, where needed, a clinical psychology intervention to support people returning to work after traumatic injury [35]. Using multiple methodologies, this paper describes and evaluates PPIE across all aspects of the ROWTATE research programme from defining research programme aims to translation of research into practice from the perspective of PPIE group members and researchers. The research programme records allow capturing this impact across 7 years. The paper is fully co‐written with PPIE contributors. It aims to describe and understand (a) PPIE contribution to a large‐scale research programme, barriers and facilitators, (b) the impact on the PPIE group members themselves and (c) the impact of PPIE on the research team.

## 2 Methodology

### 2.1 Design

A multi‐method design captured the description and evaluation of PPIE. This included (i) a purposely designed survey completed by PPIE group members and researchers, (ii) a detailed prospectively completed ‘You Said, We Did’ table and (iii) minutes from all meetings where PPIE group members took part.

### 2.2 Data Collection

The electronic survey (Microsoft Teams) was sent multiple times to all members of the PPIE group (*n* = 18) in 2024, and was completed by seven PPIE group members (39%). The survey contained open‐ended questions on PPIE contribution, barriers and facilitators and the impact on the PPIE group members themselves adapted from Mann et al. [36]. Of the PPIE responders, five were still active in the study and two had stopped engaging with key meetings. The electronic survey was also sent to eight members of research team (thereafter researchers) who worked more closely with the PPIE, and seven of them completed the survey in 2025. This included four members of the programme management team who remained members for the whole duration of the research programme and three researchers who had worked with PPIE group members very closely during the years they were employed on the project. Copies of the PPIE and researcher surveys are included in Supporting Information S1: Table S2.

The ‘You Said, We Did’ table (Supporting Information S1: Table S1), described and documented the impact of PPIE to the ROWTATE programme of research from January 2020 to September 2025. The table recorded the PPIE group members contributions and summarised how each suggestion was implemented.

The minutes of all meetings involving PPIE group members were reviewed to capture PPIE contributions. Meetings included research team meetings for each of the 4 work packages, Programme/Trial Management, Programme/Trial steering committee, training development, intervention development, toolkit development, and PPIE group meetings.

### 2.3 Analysis

All data were analysed using a qualitative narrative synthesis approach [37, 38] which brought together research evidence from diverse methodologies and data sources. This allowed an overall synthesis of key findings through analysing words and text (rather than statistical manipulation) to answer the aims of the research. Whilst narrative synthesis was developed for systematic reviews, it also allows analysing different sources of data and methodologies in complex studies [37].

Several steps were undertaken to develop and conduct this qualitative narrative synthesis which included a number of iterative activities throughout. First, the team (composed of PPIE contributors H.P.; I.A.; T.J.; S.F. and researchers B.K., R.L.; K.R.; C.M.; D.K.) discussed in detail the aims and approach that should be undertaken to describe and evaluate the PPIE contribution. Data collected during the research programme was considered. We identified minutes from all meetings where PPIE were present and the ‘You said, we did’ table as valuable sources of information that recorded information over the duration of the programme. While these discussions had been held regularly within the research programme, it was considered necessary to document this information in a format that facilitated data analysis. As a result, existing research was identified that provided the framework for additional data collection in a survey form [36]. The Mann et al. [36] method was replicated for the study and the surveys of PPIE group members and researchers. This new data collection in the latter stages of the research programme allowed asking more direct questions about the PPIE group members experiences and impact.

Next, a preliminary synthesis was developed based on the PPIE survey data to ensure the PPIE voice was given priority in the analysis. Anonymised data from the surveys were coded by PPIE contributors (H.P.; I.A.; T.J.; S.F.), then discussed as a group to agree on the codes. The coding was organised around key questions about expectations, importance, influence, impact, barriers and facilitators and impact of COVID. The main aim for this was to ensure each PPIE contributors was allocated a limited number of question each to work on before bringing the results together in the team meeting. The PPIE involvement in the coding and analysis was agreed before data collection so the team was clear that everyone would have access to the data and that anonymity could be limited because the team knew each‐other well. These were topics had been regularly discussed in PPIE group meetings where the PPIE contributors felt comfortable sharing both positive and negative comments. These key questions formed the core of the analysis which was first drafted by a PPIE contributor (H.P.). The next step included exploring the relationship and triangulating information between this core set of data (PPIE survey) and the other data collected (researcher survey, minutes from meetings and the ‘You said we did’ table). The final steps included identifying the appropriate evidence that would illustrate the key findings, and then drawing conclusions while considering the strengths and limitations of each source of data.

## 3 Results

### 3.1 Part 1: Description of Our PPIE Group

Recruitment of PPIE group members began in January 2019, ahead of the ROWTATE research programme. We aimed to ensure variation in injury type, injury mechanism, impairments, job type (part‐time, full‐time, volunteer, education) and EDI characteristics. Invitations were sent to individuals with lived experience of traumatic injury, including those involved in shaping the original research proposal in 2017, PPIE group members from previous studies led by the research team, extensive networks of co‐applicant clinicians (which included traumatic injury organisations), letters written to charitable organisations by the PPIE co‐applicant in the research grant application, and visits that the research team undertook to support groups (e.g., to recruit Black Asian and Minority Ethnic‐BAME community members). As the team reflected on the characteristics of the PPIE group members, new adverts were distributed to all contacts outlined above. For example, several efforts were made to recruit PPIE group members from BAME backgrounds. Demographic and injury details of the PPIE group are shown in Table 1.

**Table 1:** PPIE group members' characteristics.

Characteristics	Categories	Number (%) of PPIE group members *N* = 18	Number (%) of PPIE contributors to this paper (*N* = 5)
Gender	Male	10 (56%)	2 (40%)
	Female	8 (44%)	3 (60%)
Type of injury	Polytrauma	6 (33%)	1 (20%)
	Traumatic brain injury (TBI)	5 (27%)	3 (60%)
	Spinal cord injury (SCI)	2 (11%)	1 (20%)
	Limb injury	5 (27%)	0
Ethnic background	White	16 (89%)	5 (100%)
	Mixed race	2 (11%)	0
Age	18–30	1 (6%)	1 (20%)
	31–45	9 (50%)	1 (20%)
	46–60	4 (22%)	1 (20%)
	61+	4 (22%)	2 (40%)
Work status pre‐injury	FT Employed	14 (77%)	4 (80%)
	FT Self‐employed	2 (11%)	1 (20%)
	FT Education	1 (6%)	
	FT Military	1 (6%)	
Work status post‐injury	PT Employed	1 (6%)	1 (20%)
	PT Self‐employed	1 (6%)	0
	PT Voluntary	3 (17%)	1 (20%)
	FT Employed	9 (50%)	3 (60%)
	FT Self‐employed	1 (6%)	0
	Retired	3 (17%)	0
Current work status	PT Employed	4 (22%)	1 (20%)
	PT Self‐employed	1 (6%)	2 (40%)
	PT Voluntary	1 (6%)	0
	FT Employed	5 (27%)	0
	FT Self‐employed	2 (11%)	0
	Retired	4 (22%)	2 (40%)
Location of current residence	City for lead ROWTATE research site	7 (39%)	3 (60%)
	Other city	11 (61%)	2 (40%)

Abbreviations: FT, full time; PT, part time.

All those interested were invited to an initial PPIE group meeting in May 2019 (or subsequent meetings for later recruitments), introducing members to the programme, providing an opportunity to share personal experiences and helping build group rapport. One member, with prior public involvement experience and a role as a ROWTATE research grant co‐applicant, served as Chair of the PPIE group.

As planned in the ROWTATE programme design, after recruitment, all PPIE group members were invited to engage in various aspects of the research, aiming for two PPIE leads per task. Activities included monthly work package meetings, programme/trial management committee meetings, bi‐annual steering committee meetings, and development of the intervention (e.g., therapist training and implementation toolkit). PPIE group members also contributed to the delivery of the therapist training, process evaluation data collection including health economics, and publication writing (see [39] for full research programme details). Furthermore, to ensure group coherence and provide opportunity for oversight of research programme progress, PPIE group‐specific meetings were held quarterly throughout the programme, typically attended by 8–10 members. Meetings included a progress update from a chief investigator, followed by a research team presentation and discussion or an activity on a topic requiring PPIE group input.

Initially, all meetings were held in‐person at the University of Nottingham with lunch and refreshments. Sessions lasted 2 h with a break, and accessibility needs (e.g., mobility, sight, hearing) were accommodated. From March 2020, meetings moved online via MS Teams due to COVID‐19, which enabled broader geographic participation. Since early 2023, a hybrid format has been adopted.

PPIE group members were compensated at £15/h (later £25) for meetings, preparation, and additional tasks, with travel expenses reimbursed. Although 18 PPIE group members were initially involved in the research programme, by 2025, only seven remained involved, and five of these were actively involved.

### 3.2 Part 2: Evaluation of Ppie Group Members' Experience

#### 3.2.1 PPIE Contribution to the Research Programme

In the responses to the open‐ended survey questions, the ‘patient perspective’ was notably perceived to be integral to the research programme and an intrinsic part of the PPIE contribution to the research. Most PPIE group members agreed that they could provide the much‐needed insight from the patient or person with lived experience of serious injuries' perspective:

To give the perspective from a service user.

(Survey, PPIE participant H)

Helps to keep things real.

(Survey, PPIE participant G)

Provide insight into the need for appropriate rehabilitation for those affected by trauma.

(Survey, PPIE participant F)

The value highlighted by the PPIE group members includes paying attention to the role of the trauma context in providing ‘appropriate rehabilitation’. This was reflected in the researchers’ accounts:

Those with lived experience of traumatic injury have had a vital role to play on so many parts of the study—from developing the research question to disseminating the findings and all stages in between. Even before we developed the research question, discussions with one of our PPI group who was contributing to an earlier study helped me understand the importance of getting back to work from the patients perspective and helped shape my thinking about our research question.

(Survey, research participant C)

The lived experience of people surviving major trauma has helped to ensure our ROWTATE intervention is relevant for people with serious physical, cognitive and psychological injuries. The real time feedback on issues encountered in research delivery has helped to address many challenges.

(Survey, research participant D)

Evidence of the PPIE group members bringing their perspective to the research programme is also captured in the ‘You Said, We Did’ table where members described their own stories of traumatic injury. These experiences informed the training, topic guides and analytic frameworks for interviews and focus groups with trauma survivors. (see Supporting Information S1: Table S1 for further examples).

Survey responses highlighted how having lived experience ensured the patients' benefits remained a priority in the research. This included outcomes important to patients such as self‐worth, which reflects changes due to loss of valuable identities and changes due to trauma:

To represent the patient perspective. To ensure that study objectives and methods would always ‘consider patient benefit as the number one priority’.

(Survey, PPIE participant C)

PPI input was essential to guiding the study from a lived experience point of view. Because the population of ‘major trauma patient’ is so heterogenous it was impossible to capture all the possible impacts of trauma without this.

(Survey, research participant E)

For me the most important of many influences was the inclusion of ‘sense of self‐worth’ as one of the secondary study objectives.

(Survey, PPIE participant B)

This contribution to key outcomes mentioned in the last quote is captured in the ‘You Said, We Did’ table (Supporting Information S1: Table S1) where the PPIE group identified an additional key outcome—purpose in life—which was added to outcomes measured in the trial [40].

PPIE group members also believed their input provided the necessary balance between ‘researcher’ and ‘patient,’ addressing known power imbalances identified in previous research:

Instead of just professionals discussing it without other perspectives.

(Survey, participant A)

Balance the ‘research/medical’ input.

(Survey, participant G)

It's important to have the perspective of service users in the research study.

(Survey, participant H)

This was supported by the researchers’ accounts:

It is important to ground the research in the reality of the people who we want to help. They always bring us back to why we are doing the research, and ensure that the patients are our focus. It ensures that a group of educated people are not just telling patients what they need, but ensuring that we are creating something that the patient wants and needs.

(Survey, research participant F)

To provide expert guidance from the perspective of lived experience so that our approach minimised the risk of harm to the study participants, but also it ensured that researchers attended to the most important aspects of the impact of injury, not what we thought they were.

(Survey, research participant E)

Bring patient's views, help make research/intervention accessible and more applicable for the right group of patients.

(Survey, research participant J)

Keep us focused on what patients need and want.

(Survey, research participant B)

The evidence on the PPIE group members’ contributions to the research was extensive. For example, helping educate researchers on key issues such as the impact of traumatic injuries, and what contributes to patient engagement to the intervention:

Given researchers a greater insight on traumatic injuries and how they affect people.

(Survey, participant A)

provided clarity to the study team of what the patients are experiencing and allow them to understand how best to adapt the study and also understand why responses and engagement might be more challenging than expected.

(Survey, participant F)

Again, there is a strong emphasis in making a positive contribution that not only provides ‘a greater insight’ but also helps anticipate challenges which are due to the context of trauma recovery. Researchers also recognised this extensive contribution which expanded and developed with time:

Group members have had many roles in the study and have taken on roles that weren't initially envisaged for them at the start of the study. They have contributed to (1) shaping the research question, (2) selecting the outcome measures for the study and the tools to measure those outcomes, (3) developing all patient facing documents for the study—questionnaires, information sheets, consent forms, qualitative interview guides and the patient pages on the study website, (4) developing recruitment and retention strategies for the study, (5) training OTs and CPs to deliver the intervention, (6) interviewing study participants, (7) identifying employers for study interviews, (8) drafting study information and recruitment strategies for employers, (9) analysis of interview data, (10) study management and oversight via trial and programme management groups, (11) writing for publication, (12) drafting PPI sections for reports to funders and (13) study oversight via the Programme Steering Committee.

(Survey, research participant C)

Contributing all the way through from ideas to data collection tools having a say on analysing and interpreting data especially quali data and patient voice and through to dissemination and writing.

(Survey, research participant A)

Another example captured in the ‘You Said We Did’ table (Supporting Information S1: Table S1), related to the health economics impact. Whilst some health economic studies consult closely with PPIE representatives, the ROWTATE research programme further utilised a specific health economic PPIE focus group. Assessing whether the intervention offers value for money to the taxpayer is a core requirement of NIHR‐funded research. Drawing on the insights of individuals with lived experience of trauma and their engagement with NHS services is critical in capturing costs. A PPIE focus group explored key sources of health care resource use (inpatient, outpatient and community care), and costs to the individual. PPIE group members also advised on suitable ways to accurately record service use, medication, and personal costs. They suggested a calendar diary or a smartphone app could be helpful. Based on this feedback, a paper‐based resource use monthly wall calendar was developed, as many PPIE group members noted difficulties using smartphone applications. PPIE group members were also consulted on question clarity and wording, questionnaire burden, how to improve completion rates and how to overcome challenges with missing or incomplete health economics data.

PPIE group members also provided feedback on all patient‐facing materials (information sheets, questionnaires etc.) prior to the feasibility study and clinical trial. All study materials were updated in accordance with PPIE group feedback (Supporting Information S1: Table S1). Finally, PPIE group members also assisted in collecting interview data for the process evaluation part of the research programme.

PPIE group members and researchers outlined the PPIE contribution towards ensuring appropriate communication with patients or avoiding miscommunication. This was considered vital for informing and retaining both patients along with PPIE group members.

It is essential that patients are contacted with and communicated to appropriately for their condition and issues with their recovery.

(Survey, participant F).

It (PPIE contribution) has improved the study in multiple ways. It has contributed to the success of our study in terms of recruitment and retention, helped us measure outcomes that are meaningful to people with lived experience of trauma, allowed us to incorporate the views of those with lived experience into our intervention as well as into our analyses and interpretation of our data and kept our focus on what matters to patients.

(Survey, research participant C)

The PPIE group members and researchers believed that PPIE not only shaped and directed the trajectory of, but also made direct contributions to the research programme and intervention.

We have been able to directly input into the delivery of the intervention.

(Survey, PPIE participant B)

For me their role in intervention and training development and training delivery was the most important part. I also believe their sustained reflection on all research activities and contribution to deliverables helps us to remain grounded and reflect what we do/have achieved against the intended purpose.

(Survey, research participant D)

The patient video was very powerful probably the most impactful contribution and I hope they can continue to help us do more of this.

(Survey, research participant A)

The full range of the PPIE group's contributions is further noted in the ‘You Said, We Did’ table (Supporting Information S1: Table S1). PPIE group members were able to bridge the divide between the academic and ‘real world’ by presenting their real‐life experiences to provide researchers with a ‘sounding board’ and a ‘reality check’. The PPIE group members were also able to challenge the approach and methodology of the programme and researchers and felt able and encouraged to voice any concerns or improvements that could be introduced. The feedback from the PPIE group members was that they were being heard and listened to, which had a direct and positive impact on the research programme. Additionally, PPIE group members were part of a working group developing the therapist training package. These contributions informed development and delivery of training materials subsequently used to train therapists to deliver the ROWTATE intervention (this is captured in more detail in [41]).

#### 3.2.2 PPIE Group Members’ Understanding of Their Role

A primary reason for getting involved was to help others through their own recovery journey:

Hopefully help someone like me get back into education.

(Survey, PPIE participant A)

Help other people who have suffered traumatic injury receive the same kind of return to work support that I benefitted from.

(Survey, PPIE participant C).

In terms of the long‐term impact of the research programme, PPIE group members reported their motivation to bring about sustainable change for employers, but also to shape the way patients are supported in the future.

To improve things for future patients and empower employers to gain more knowledge and support their employees who have a traumatic injury. Open a dialogue around how best to help their employee…. to make a difference.

(Survey, PPIE participant A)

Organisations welcoming employees who have suffered major trauma, back to the workplace in a planned and effective way.

(Survey, PPIE participant G)

Make a positive change to hospitals and treatment.

(Survey, PPIE participant E)

When asked in the survey, most of the PPIE group members were clear about their contributions to the research programme. These answers often reflected their unique roles, such as contribution to the training, development of research programme documents, involvement in the trial and so on.

PPIE members have been actively encouraged to engage and contribute ‐ I have always felt that voices have been listened to and diversity respected. Leaders of the study have been open to ideas, suggestions etc.

(Survey, PPIE participant G)

To the therapist's training sessions.

(Survey, PPIE participant B)

This clarity of PPIE contributions was evident in all the research team responses, who were able to identify PPIE contributions in detail. This is evidenced in many of the quotes in the previous section and illustrated further below.

Their lived experience helping design and implement the research and intervention…Every aspect of the research.

(Survey, research participant J)

However, some of the PPIE group members did not feel they had a consistent understanding of their contribution due to miscommunications between the research team and the PPIE group members, or lack of adequate feedback and information given throughout the research programme.

I'm not sure, in the end, what value there was in my input. There was very little feedback.

(Survey, PPIE participant D)

…perhaps more PPIE contributions need to be highlighted in meetings or in newsletters?

(Survey, PPIE participant A)

These responses reflected the experiences of two of the PPIE group members who had not remained active in their role at the time of the data collection. It is also likely that the long timelines of the ROWTATE research programme (7 years) might have further contributed to this poor understanding as, for example, many aspects of the research programme were designed months and even years before they were carried out. Some planned meetings also did not take place due to challenges with recruitment as is the case of the employer meeting mentioned in the next response:

In the last 9 months it has felt that the project has lost a bit of traction.  I was asked to contribute to an employer's meeting but have heard nothing since.

(Survey, PPIE participant G)

These later responses show that not all PPIE group members felt that their input was clear in the research programme, calling for diverse methods and more frequent communication. This is an issue which will be explored further in the discussion. Nevertheless, all PPIE group members were able to give at least one example of the groups contribution in terms of importance of the patient perspective particularly to balance with researchers, shaping delivery and facilitating patient communication throughout.

#### 3.2.3 Benefits and Value for PPIE and Research Team

The PPIE group members clarified that when joining the research programme, they felt that bringing their own perspective to the research programme contributed to their sense of self‐worth and value.

Making a difference and have my ideas and opinions valued and implemented… ‘Being appreciated for my contribution.’

(Survey, PPIE participant B)

Knowing that our experiences have helped to inform the research study.

(Survey, PPIE participant H)

Yes, it is very rewarding and makes me feel like I have a purpose (in) life following my accident.

(Survey, PPIE participant E)

One PPIE group member explained how they gained personal satisfaction from being part of clinicians’ training sessions as they felt they could give them real practical insight.

Attending OT training sessions online and in person. Mainly because they got to have an idea of the people and practices they would be working with.

(Survey, PPIE participant A)

Learning about the positive PPIE group contributions was also beneficial:

…The therapists in the therapists’ training sessions have feedback and said to us directly what a positive difference it made having PPIE members involved.

(Survey, PPIE participant B)

Knowing that my experience has helped with the study.

(Survey, PPIE participant H)

Part of the benefits included PPIE group members’ intellectual contributions and development:

Kept my brain active. Given me an insight in how studies work and how they are implemented.

(Survey, PPIE participant A)

‐ Sense of continuing to do useful service to others in my retirement… Keeps my brain active!!

(Survey, PPIE participant C)

Another key value was the opportunity for PPIE group members to learn more about their own injury and personal growth, and to meet with others who have a shared experience which contributed to a sense of community:

Provided more insight into how medical studies are undertaken and also meeting other PPIE individuals who have experienced similar challenges and experiences to myself.

(Survey, PPIE participant (F)

It has been very positive and has helped me personally accept what has happened to me in coming to terms with the effects of my TBI and disabilities. And to use my experience to help others is very rewarding.

(Survey, PPIE participant E)

It has been great to meet others who have survived major trauma, hear their experiences which are all different and inspirational.

(Survey, PPIE participant G)

The value of involvement was also clear in some responses to the survey question of whether they would consider any future PPIE group involvement:

Definitely, I feel it has helped me personally and I feel that I have been able to make contributions and be engaged with the others and also the study team.

(Survey, PPIE participant F)

Yes, I would welcome the opportunity to contribute again if my input would be of value.

(Survey, PPIE participant G)

More likely as it's been a really positive and worth‐while experience.

(Survey, PPIE participant B)

Finally, when asked what they hoped to get out of their involvement, PPIE group members highlighted diverse reasons such as: sense of purpose, recovery of personal skills, but also the benefits highlighted in earlier in Part 2 such as helping future traumatic injury patients through their recovery journey, and bringing a patient perspective to the research programme team.

Gain new experiences and get my brain working again…

(Survey, PPIE participant A)

Do something meaningful with my time… Hard work…. but mentally stimulating, cognitively and mentally demanding.

(Survey, PPIE participant B)

Sense of valuable contribution.

(Survey, PPIE participant D)

The opportunity to contribute and shape the outcomes.

(Survey, PPIE participant G)

One PPIE group member was less positive about their contribution because they felt they had received no feedback on their individual or group contributions to the research programme. The updates provided in PPIE group meetings were not successful in informing this PPIE group member of their valuable contribution to the research programme.

Vaguely positive. I've had no feedback, either personally or as a group.

(Survey, *PPIE* participant D)

This was recognised by one of the researchers:

Keeping track of research which can move back and forth and sideways. I think it was only about 4 members of the whole team people who were involved in everything. Most other members of the research team worked on specific work packages as did the PPI.

(Survey, research participant J)

The research team also reflected on how working with PPIE group members impacted on their understanding as well as motivation for working on the programme. When asked what has given them the greatest satisfaction of working with PPIE group members they reflected on the valuable, inspiring and positive role of PPIE:

Getting to know them as human beings and recognising how important their contribution to research was to them personally. Having lost quite a lot through injury, their motivation to renew their own sense of purpose while simultaneously aiming to make the world a better place for the next generation of injury survivors was really inspiring.

(Survey, research participant F)

Knowing that not only are they helping the study, but knowing that their work on this study has helped them. Either by connecting them with fellow injury survivors or using this as their own ‘vocational rehabilitation’ to help them get jobs. The positive impact that working on the study has had for the PPI has given me the greatest satisfaction.

(Survey, research participant G)

The positive mindset that people with life changing injuries bring to the project… I have really enjoyed working with the PPI throughout my time with ROWTATE. I really appreciate their time and commitment to the study. They have made my job a lot more interesting by being part of it.

(Survey, research participant D)

I really enjoy working with the PPI group members and it is great to see the really positive impact they have had on the study.

(Survey, research participant C)

For PPI to see how their contribution changed our work.

(Survey, research participant J)

Two of the research team members felt that even their career was positively influenced by working with PPIE group members:

The PPI groups willingness to share their lived experience so candidly directly influenced my own career development because it was inspired my subsequent PhD thesis idea which was built upon their qualitative interview data. I felt like I really gained some deep insight into the challenges they overcame and wanted very much to do justice to their sharing of highly personal experiences. I think I learnt also quite a bit about how to plan for inclusivity where people are dealing with a range of impairments.

(Survey, research participant F)

They motivate me to continue working on the study, reminding me of the reason we work on this study and importance of vocational rehabilitation for traumatic injury survivors. They make me consider my own work and whether it is accessible for lay people. It also brings me joy to work with them, I have really enjoyed working with them and it has been one of my favourite parts of my job.

(Survey, research participant G)

#### 3.2.4 Facilitators to PPIE Group Members Engagement

In terms of facilitators to PPIE group members’ engagement, participants listed the flexibility and variety of tasks available in the research programme, and the professionalism and willingness of staff to make reasonable adjustments.

Ironically not being in work at the time and also the flexible to engage remotely as well as face to face.

(Survey, PPIE participant F)

And the range of roles/tasks available. The flexibility by (name of researcher) and other staff and the reasonable adjustments made for online and in person sessions.

(Survey, PPIE participant B)

MS Teams—it would have been a real challenge to meet onsite due to the travelling distance.

(Survey, PPIE participant G)

The research team also supported the value of these facilitators and reflected on the importance of building positive and close relationships with the PPIE group.

Building close relationships with the PPI so they feel supported to fulfil their potential, as it has allowed them to feel comfortable coming to me to ask for help or make suggestions. Having other staff on the trial who also appreciate the contribution of the PPI and want to facilitate it more. Having an understanding of the PPI's needs and how we can help them be involved as much as they want. Having lots of different ways PPI can be involved in the study, so they can be involved as much as they want, in what they are interested in. Having very motivated and engaged PPI members, who care about the study, and are keen to be involved with as much as possible to make a difference.

(Survey, research participant E)

It helps building personal relationships and responding to their needs and very rightful adjustments.

(Survey, research participant J)

The length of the research programme has enabled us to build really good relationships between the PPI group and the research team…It needs commitment and continuity and the investment of one person to lead and manage it.

(Survey, research participant C)

The transition to online and hybrid meetings after the pandemic was considered to have had little bearing on the delivery of meetings both from PPIE group members’ and researchers’ perspectives.

Meetings aren't really affected much by the hybrid nature.

(Survey, PPIE participant A)

Group adjusted well to changes.

(Survey, PPIE participant H)

I think having remote/hybrid meeting has helped with accessibility for some group members, but possibly not for all. Long meetings can be difficult for some group members but having breaks can help this.

(Survey, research participant C)

In fact, the hybrid nature of the meetings meant that PPIE group members did not have to travel which allowed for a greater geographical spread of members. Also, it reduced the pressure of those who had impairments because of their traumatic injury.

Takes pressure off if you can't face travelling (due to fatigue).

(Survey, PPIE participant A)

Enables greater geographical spread—group had been too Nottingham centric.

(Survey, PPIE participant C)

Other PPIE group members referred to practical facilitators which were specific to their situation as well as personal experience as a reason for taking part.

(Getting) lifts from my parents to In‐person meetings

(Survey, PPIE participant A)

Professional background in business management means that I am used to playing a leading role in management teams.

(Survey, PPIE participant C)

Researchers also reflected on these personalised facilitators and their importance:

Helps that we pay and make every accommodation requested. (Survey, research participant A)

Having a glossary of terms and avoiding abbreviations and acronyms has been helpful, although we do sometimes forget!

(Survey, research participant C)

Some of the facilitators to PPIE are captured in the ‘You Said, We Did’ activity log. For example, the PPIE group members suggested the reduction of the number of acronyms, regular breaks during meetings to maintain focus, reduced frequency of meetings to quarterly to allow focus on key issues, and training of PPIE group members on the programme activities. In response, the research team created a glossary of terms, provided training which included explanation of trial methodology and terms, and captured more systematically and provided feedback on PPIE group contributors’ suggestions. Detailed notes of PPIE group meetings were written and circulated to all attending and for researchers to check for accuracy. The meeting notes were sent to the whole PPIE group, who were also asked to amend anything they felt to be inaccurate. Also, it was felt that areas where PPIE group members could be involved in the research programme should be clarified. As a result, a table showing suggested activities in which PPIE group members may be involved during the main trial was produced and circulated. A key link from the research team and a PPIE group lead were appointed and the PPIE group were made aware that they could contact the Chief Investigators at any time should they wish.

#### 3.2.5 Barriers to PPIE Group Members Engagement

Almost all the barriers identified by PPIE group members in the survey shared a commonality pertaining to impairments because of their traumatic injury, including brain injury. For instance, fatigue and sometimes comprehension have prevented members from fully engaging in the research programme at times.

Sometimes the travel was challenging as well and also at times remotely when lots of talking and discussions take place.

(Survey, PPIE participant F)

The effects of my brain injury means I sometimes have difficulty in understanding certain things.

(Survey, PPIE participant E)

The research team members also reflected on this key context of PPIE experience:

Our PPI were grappling with their own impairments, which at times limited the extent to which they could contribute. Fatigue and mobility in particular were barriers that we had to make efforts to overcome by planning interactions that accounted for their impairments sensitively.

(Survey, research participant F)

Remote meetings could be challenging and at least two participants preferred in person meetings:

I prefer face to face.

(Survey, PPIE participant D)

I always preferred the meetings in person.

(Survey, PPIE participant E)

More challenging to actively engage at time(s).

(Survey, PPIE participant F)

Not always easy to engage remotely.

(Survey, PPIE participant G)

Retention of the PPIE group members with diverse experiences over time was also highlighted as a potential barrier in the survey by PPIE and research team:

Need to attract, or retain, a larger and wider PPIE group.

(Survey, PPIE participant D)

Could have been more diverse though that is not for lack of effort.

(Survey, research participant B)

In fact, extensive efforts were made to increase the diversity (injury types, age, ethnicity and geographical spread) and number of PPIE group members via circulation of advertising to personal contacts, patient interviews and communication with the Centre for BME Heath in Leicester, social media, and display of posters in hospital clinics (see Supporting Information S1: Table S1 for further examples).

For one of the PPIE group members, especially, there could have been better communication in and between meetings.

In case I haven't made it clear, there needs to be more feedback… Needs to be more focus on PPIE in specific meetings without the internal admin…. I am involved in a second study. My contribution is much more obvious, and the meetings are more focused on getting our feedback on specific decisions being made. The ROWTATE meetings were often lengthy and only occasionally asked directly for PPIE Feedback.

(Survey, PPIE participant D)

This participant calls for even more PPIE group‐specific meetings (in addition to the 3–4 per year) which do not focus on research programme specific administration. The same PPIE group member who praised adjustments made by the research programme team to PPIE group involvement, also highlighted the lack of adjustments at times which were presented as a barrier. This suggests that while broader adjustments can easily be accommodated these need to be consistent throughout.

The lack of adjustments at times.

(Survey, PPIE participant B)

The research team changes and retention as well as the nature of work on the project could have also been an additional barrier:

It (working with PPIE) needs commitment and continuity and the investment of one person to lead and manage it. This offers a great learning opportunity for research fellows and research assistants but sickness absence and staff changes sometimes meant contact and continuity was fractured and a bit hit and miss.

(Survey, research participant D)

Due to part time work I have not been able to attend all the PPI group meetings.

(Survey, research participant C)

As illustrated in the ‘You Said, We Did’ table, it was felt by some PPIE group members that communication with the PPIE group should be clearer with full opportunity for discussion and feedback around key issues. In response to this feedback, PPIE group meeting dates, times agendas and key documents were circulated in advance of PPIE group meetings, and regular meeting breaks were introduced to help maintain focus during meetings. (see Supporting Information S1: Table S1 for further examples).

The researchers also reflected on the nature of some of these barriers including the challenges of implementation of some of the suggestions from PPIE and ensuring adequate communication and collaboration and number of items in the meetings’ agendas:

Balancing the suggestions from the PPI with the needs of the trial, as they don't always match up. They also often have suggestions that cannot be fulfilled within the confines of this study. It can also be challenging blending lay people into the research team, as they is a lot of technical language to be understood and they require additional support to have an understanding so they can make relevant contributions. Sometimes it is challenging when PPI have differing opinions or suggestions. Working with a group of predominantly brain injury also has unique challenges, that not all members of the research team are as adapt at handling. Working with the PPI can be very time consuming (although usefully time consuming), which is not always appreciated by all members of the research team, leading to pressure to meet commitments. …I also think there is frequently far too much on the agenda and an overreliance on MS Teams meetings that are short, with no time for discussion and very little reflection with PPI in real time on how their contribution has/is making a difference.

(Survey, research participant D)

Some of the challenges were more institutional:

I think the most challenging thing is finding the time to do this as well as I would like personally and for the study. Then there have been numerous barriers to involvement for example, the employment related and occupational health checks required for the research passport to enable the PPI members to be involved in interviewing participants. We also wanted to consider PPI involvement in recruitment and ‘peer support’ as a bolt on to the intervention but the need for DBS checks and payment for these, plus the Sponsor's risk averse governance processes meant this was not an option.

(Survey, research participant C)

Hinders that the uni system means payments are slow and in vouchers which PPI don't like and I think prevents some people from participating.

(Survey, research participant A)

## 4 Discussion

### 4.1 Summary of Key Findings

This paper aimed to describe and evaluate the role of PPIE in a research programme that included a clinical trial, and barriers and facilitators to PPIE through a mixed method design (PPIE survey, Researcher survey, ‘You said, we did’ table and meeting notes). The findings provide a multi‐perspective evaluation of the role, experience, and impact of PPIE within a large‐scale, longitudinal research programme focusing on vocational rehabilitation and clinical psychology following traumatic injury. The findings show that PPIE group members played a significant and multifaceted role throughout the research lifecycle, from research design and intervention development to data collection, analysis, and dissemination. The involvement of individuals with lived experience enriched the research process by ensuring that the patient perspective remained central, thereby enhancing the relevance, accessibility, and potential impact of the research programme. Psychologically, PPIE became also a valuable and meaningful experience, a type of group membership that has been shown to be very beneficial when dealing with traumatic change and loss of valued identities [28, 42].

Analysis of survey responses revealed that PPIE group members were motivated by and felt they could bring the patient voice into the research programme, help others with similar experiences, and contribute to meaningful change in healthcare practice. From both PPIE and researchers’ perspectives, PPIE contributions were perceived as instrumental in shaping research design, materials, improving communication strategies, and informing the training of clinical staff. Importantly, PPIE also had a positive personal impact on PPIE group members, fostering a sense of purpose, intellectual engagement, and community, some of which were also reflected in the research team's own experiences of their work. Again, this type of activity can be very beneficial when facing difficult life changes [43]. Helping others and bringing sustainable change are actions that reflect embracing a new identity that enhances self‐worth and can be a key strategy to deal with change and trauma [28]. Furthermore, this focus on meeting others who share the same experiences, is also recognised as a key strength when dealing with change and trauma [28, 44].

However, the evaluation also identified several challenges some of which reflected those of previous research [45, 46]. These included retaining PPIE over time (only five PPIE remained very active at the end of the study, and two engaged rarely) and engaging with PPIE group members more inclusively, despite multiple attempts. Some PPIE group members expressed uncertainty about the value or visibility of their contributions, citing limited feedback and communication as barriers. The long duration of the research programme (7 years in total, impacted by the COVID‐19 pandemic) and distribution of the PPIE group across different tasks may have compounded these issues. Working on complex programmes of research like ROWATE can be difficult to explain, and working on one part can make it difficult to grasp the whole programme which develops multiple workstreams at the same time. The researchers reflected that on some occasions the PPIE ideas suggested did not fit with the programme of research or took longer time to realise, potentially leading to disillusion about what research can do in the shorter versus longer term. Equally it is possible that PPIE work was valuable short‐term while PPIE group members were recovering from their injuries and then were able to take up other more substantial employment.

When reflecting on these issues, PPIE group members suggested that a potential contribution to lack of clarity and retention could be due to the cognitive impairment and memory difficulties that several members of the PPIE group experienced as a result of their traumatic injury. This suggests that paying attention to specific research context and nature of lived experience is important because it can contribute to the complexity of understanding and evaluating PPIE involvement and maintenance over time. One such specific context is that of trauma which has several unique features. Traumatic events can have long lasting physical and psychological impacts leading to long‐term disability and transformed social relations [47], which can impact the survivors ability to engage with health services [26] and research [48]. To address all these challenges is resource intensive and requires commitment and skills and experiences on the part of the research team. However, the positive and meaningful experiences reported showed that PPIE can be a valuable group membership and activity to support recovery and life transition following injury [28]. Researchers also reflected on the importance of building relationships and consistent communication which did required important changes to how meetings were organised and structured.

### 4.2 Contribution to Theory and Practice

This evaluation contributes to the growing body of literature on PPIE [5, 6, 7, 8]. However, this evaluation offers a multi‐perspective account of involvement in a complex, multisite programme of research providing novel insight into vocational rehabilitation [49] and clinical psychology research and practice [50]. It underscores the importance of embedding PPIE not only as a methodological enhancement but also as a democratic imperative that values the experiential knowledge of patients and the public. From a theoretical perspective, the findings support the conceptualisation of PPIE as a dynamic, personalised and context‐dependent process (in this case trauma) that requires ongoing negotiation, support, and adaptation [1, 51]. It also supports key principles of successful PPIE which include PPIE skill development and confidence, person‐centred approaches, developing trust and building relationships between researchers, the PPIE group and communities [25], some of which are key to successful recovery from trauma and its related life transitions [28, 44].

The evaluation, however, recognises both positive aspects and challenges and highlights the need for clear role definitions, consistent communication, and mechanisms for feedback to ensure that PPIE contributors feel valued and informed. Practically, the evaluation offers several recommendations for future research involving PPIE, such as inclusive recruitment of diverse contributors with lived experience, structured support and training to enable meaningful involvement, regular, transparent communication about study progress and the impact of PPIE input, flexible engagement formats that accommodate contributors’ needs and preferences.

### 4.3 Strengths and Limitations

A key strength of this evaluation is its collaborative approach, with PPIE group members actively involved in the design, implementation, and authorship of this evaluation. This enhances the credibility and authenticity of the findings. The use of multiple data sources, including surveys, meeting minutes, and the ‘You Said, We Did’ table, allowed for triangulation and understanding of PPIE impact over time.

Another strength of this evaluation lies in its distinctive inclusion and clear reporting of PPIE in both the design and implementation of vocational rehabilitation, clinical psychology and the health economics component. While PPIE is becoming more embedded across health research, its application within vocational rehabilitation [49], clinical psychology [52, 53] and health economics [54, 55, 56] remains under reported, with limited evidence on how patients have been meaningfully involved and what impact this has had. Health economics especially is frequently perceived as technical and detached, which can present barriers to involvement [54]. Nevertheless, our findings suggest that actively involving PPIE group members as valued research partners can improve the relevance and understanding of economic data collection tools, and helps ensure that research programme outcomes are more closely aligned with the priorities and lived experiences of those affected. Another key strength is the team of experienced researchers, some of whom also share experience of trauma, bridging some of the gap between researchers and PPIE group members. Use of theoretical frameworks that capture the impact and responses to trauma and its related life transition in relation to PPIE is also a strength. Finally, the PPIE group represented a sample with diverse injury types and impairments which reflects the novelty of our programme of research, the first to develop and deliver a vocational rehabilitation intervention to patients with a diverse range of injuries [39].

However, several limitations must be acknowledged. Although the sample size (*n* = 18 for the PPIE group), is larger than that in previous research (e.g., ranging from 1 to 10 [10, 14, 16, 17, 57, 58, 59]), just over half did not remain active in the final year of the programme. Only 7/18 PPIE group members completed the survey and responses may not fully capture the diversity of experiences within the group. Among the seven who completed the survey, five were active members and two were less engaged providing a more limited understanding of the wider group. However, the other sources of data allow capturing the contributions of the other PPIE group members. The long duration of the research programme may have affected continuity and retention, and some PPIE group members’ work demands and impairments may have influenced their ability to engage consistently. Additionally, while the ‘You Said, We Did’ table provides valuable insights, it may not capture all informal or less visible contributions. A further limitation is the small number of minoritised group members in the PPIE group, while recognising that minority groups face additional complex and systemic barriers to healthcare [29]. Here the role of the PPIE is essential, in not only ensuring representation but also considering different impacts on diverse groups of the community [25]. In addition, the evaluation does not evaluate the potential costs and benefits of the time, resources and funding spent on PPIE. However, the very positive experiences highlighted by the research team members in terms of not only the value of PPIE contribution to the research and intervention but also on the research teams own work and motivation is an indication of the benefits of PPIE in this research programme. Future research should address these limitations.

## 5 Conclusions

This evaluation shows that meaningful PPIE has the potential to significantly enhance the quality and relevance of health research, particularly in complex areas such as vocational rehabilitation, clinical psychology and health economics. While challenges remain, particularly around communication, role clarity, and sustained engagement, the benefits for both research and contributors are substantial. Future studies should prioritise inclusive, well‐supported, and transparent PPIE practices to ensure that the voices of those with lived experience continue to shape and improve health research.

## Author Contributions

**Blerina Kellezi:** conceptualisation, funding acquisition, methodology, data analysis writing, supervision, writing, reviewing and editing. **Heather Peacock:** conceptualisation, methodology, data analysis writing, writing, reviewing and editing. **Trevor Jones:** conceptualisation, methodology, data analysis writing, writing, reviewing and editing. **Ali Gibson:** conceptualisation, methodology, data analysis writing, writing, reviewing and editing. **Isabel Andrews:** conceptualisation, methodology, data analysis writing, writing, reviewing and editing. **Steve Fallon:** conceptualisation, methodology, data analysis writing, writing, reviewing and editing. **Rebecca Lindley:** conceptualisation, methodology, data analysis writing, supervision, reviewing and editing. **Kate Radford:** conceptualisation, funding acquisition, methodology, supervision, reviewing and editing, reviewing and editing. **Kay Bridger:** supervision. **Claire Mann:** reviewing and editing. **Cristina Roadevin:** writing. **Marilyn James:** funding acquisition, reviewing and editing. **Denise Kendrick:** conceptualisation, funding acquisition, methodology, supervision, reviewing and editing.

## Ethics Statement

This is a project evaluation that did not require ethical approval for the collection of the data analysed in the evaluation.

## Conflicts of Interest

The authors declare no conflicts of interest.

## Supporting information


**Supporting Table 1:** “You Said, We Did” Activity Log documenting impact of PPIE input to the ROWTATE programme of research. **Supporting Table 2:** PPIE and researcher questions informed by Mann et al. (2018).

## Data Availability

The data that supports the findings of this study from the ‘You said, we did table’ are available in the supporting material of this article. For the survey materials, there is no permission from the participants to share the data.
