# Supplementary material for: Public and Patient Involvement and Engagement in Clinical Trials: A Multi‐Perspective Mixed‐Methods Evaluation of the ROWTATE Programme
Source: Health Expect. 2026 Mar 12;29(2):e70636. doi: 10.1111/hex.70636 (PMC13080889; doi:10.1111/hex.70636)
Supplement: Supplementary file 1 — Supporting Table 1: “You Said, We Did” Activity Log documenting impact of PPIE input to the ROWTATE programme of research. Supporting Table 2: PPIE and researcher questions informed by Mann et al. (2018). [file HEX-29-e70636-s001.docx]

**Supplementary Table 1: “You Said, We Did” Activity Log documenting impact of PPIE input to the ROWTATE programme of research.**

| **Date** | **You Said** | **We Did** | **UK standards for Public Involvement in Research – Approaches and behaviors designed to improve PPIE in research** |
| --- | --- | --- | --- |
| May 2019 | PPIE group members described own stories of experiences after traumatic injury | - Experiences informed the design of topic guides and analytic frameworks for interviews and focus groups with trauma survivors. | - Working together |
| July 2019 | PPIE focus group prioritised key Return to Work (RTW) intervention outcomes of importance to trauma survivors. | - Added ‘sense of purpose’ to ROWTATE secondary outcome list | - Working together - Impact |
| August 2019 | PPIE focus group discussed health economic outcome measures and provision of data collection tools to participants, highlighted issue of remembering healthcare appointments | - Developed participant diaries and A$ chart for recording of key economic data. - Updated health economic data collection tools based on PPIE recommendations. | - Working together - Impact |
| July-December 2019 | PPIE group representatives were part of a working group developing therapist training package. | - Informed development of training materials and methods subsequently used to train therapists to deliver the ROWTATE RTW intervention. | - Working together |
| November 2019 | Completed all patient facing materials (information sheets, questionnaires etc.) prior to use in feasibility study to provide feedback on comprehension and length. | - Updated materials in accordance with PPIE group feedback | - Working together |
| Jan 2020 | Patient and public involvement (PPI) Involvement needs to be increased, so as to widen the range of injury types, age, ethnicity and geographical spread. | - ROWTATE grant application included establishing a group of ten PPIE members. - Original group membership comprised 11 participants but biased towards Nottingham and TBI. - Efforts to increase number and range of members have included personal contacts, patient interviews and communication with the Centre for BME Heath in Leicester. - More recent production of recruitment poster, circulated widely through social media, Centre for BME Health and display of poster in hospital clinics. - PPIE Group membership now stands at 17 with greater diversity. Number has fluctuated over time. - Effort to further increase diversity were ongoing till end of December 2024. | - Inclusive Opportunities |
| Jan 2020 | Communication with the PPIE group should be clearer with full opportunity for discussion and feedback around key issues. | - PPIE group meeting dates and times circulated well in advance. - Separate pre-meeting email including agenda and asking group members to focus on key documents in advance of meetings. - Each PPIE group meeting includes updates from joint CIs (DK or KR) with opportunity for discussion and feedback. - Meeting frequency reduced to quarterly to allow greater focus on key issues. - Regular meeting breaks to help maintain focus during meetings. | - Communications - Working together - Impact |
| Jan 2020 | Establish central point of contact for PPIE Group members with issues or concerns. | - Key link people from the professional research team appointed (RL) and PPIE lead appointed (TJ) - PPIE group members made aware that they can contact the CIs (DK & KR) at any time should they wish | - Inclusive opportunities - Communications |
| Jan 2020 | Avoid abbreviations and acronyms. | - Created a glossary of terms, written in plain language. Glossary update as trial goes along | - Inclusive opportunities - Communications |
| Jan 2020 & July 2020 | Provide training to PPIE group members for programme activities | - Introductory meeting with RL, TJ for new PPIE Group members. - Public facing website [www.rowtate](http://www.rowtate/).org contains study information. - Bespoke training provided in semi-structured interviewing and data analysis for PPIE interested in undertaking these roles. | - Support and learning |
| March 2020 | Importance of PPIE involvement in all parts of the Study programme | - PPIE group members included in management teams for WP2 (feasibility study), WP3 (main trial) and WP4 (implementation workstream) | - Governance |
| March 2020 | Clarify where PPIE group members can be involved in study activities. | - Produced a table showing suggested activities in which PPIE group members may be involved during the main trial (WP3). - Regularly circulated the table for updating. | - Working together |
| May 2020 | PPIE group members to produce regular ROWTATE newsletters | - PPIE group members produced quarterly newsletters for circulation to whole research programme team | - Impact |
| July 2020 | Highlighted the importance of restoring a’ sense of purpose’ to people as they recover from serious injury. | - Included a measure of sense of purpose as an outcome in the main trial. - Included restoration of sense of purpose as a key study finding in published papers. - When shortening the follow up questionnaires to increase return rate made sure to keep this measure, albeit as different shorter version | - Working together - Impact |
| July 2020 | PPIE group members’ involvement in interviewing patients. PPIE group members considered patients may feel more comfortable being interviewed by people who have experienced trauma.  Discussed potential for issues arising to affect PPIE group members and how they could be supported in this role. | - Developed interview debrief for PPIE group members after every interview. - PPIE group members planned to do interviews in WP2 (feasibility study) but this did not prove possible due to delay in obtaining required permissions from hospital trusts. - PPIE involved in the interview analysis. - Researchers applied for permission well in advance of main trial interviews. - PPIE group members trained to conduct interviews for WP4 - PPIE group members conducted WP4 patient interviews | - Working together - Impact |
| Aug 2020 | PPIE involvement in the training of Occupational and Psychological Therapists who will deliver the intervention, to provide trauma survivors’ perspectives | - Four PPIE group members contributed to training development meetings. - Two PPIE group members were involved in the training of therapists for the Feasibility Study. - PPIE representatives have attended all of the therapist training that has happened so far for WP3. PPIE group members have greatly added to the training by bringing in the real life perspective, that is appreciated by the therapists. - PPIE group members also attend all refresher training and engagement events with therapists | - Working together |
| August 2020 | PPIE group members stressed the importance of having some face to face contact between therapist and participant to build rapport | - Based on this, alongside findings from WP2, intervention has a blended approach to delivery in WP3, where therapists can see participants face to face if they feel it is clinically necessary. | - Impact - Working together |
| Summer 2020  and 2021 | Provided feedback on acceptability of questionnaires produced for WP2 and WP3 | - Questionnaires were modified to take into account PPIE group members’ suggestions. This includes adjusting the health economics questions by removing some questions and adding examples for each category (e.g. primary and secondary care, time off work) | - Impact - Communications |
| Oct 2020 | Discussed and agreed follow up methods to be used in WP3, including consideration of potential participant burden and experience from previous studies. | - Follow up procedure specified in successful ethics committee application. Chief investigator reported it had been very helpful in the ethics committee meeting to report on the PPIE involvement in designing the follow up procedure. | - Impact - Communications |
| Jan 2021 | PPIE group members suggested the use of short videos in ROWTATE communications | - Discussed with PPIE group members where videos could best be incorporated into programme activity. - Identified use of video in training of staff who will be responsible for recruiting patients during the main study. - PPIE group members created videos to explain importance of ROWTATE for the website | - Governance - Communications |
| February 2021 | Provided feedback on participant information sheet (PIS) for main trial to ensure it doesn’t read as if only those with psychological issues should take part in the study after 7/10 participants in the feasibility study were referred to psychologist. | - PIS modified based on PPIE group’s suggestions for wording, was accepted by ethics, and is being used for patient recruitment - Smaller percentage of patients being referred to psychologist in main trial compared to feasibility study. Similar to original calculations of number of patients needing psychological support | - Impact - Communications - Working together |
| May 2021 | PPIE group had a consultation regarding the prioritising outcomes paper and provided feedback | - Paper modified based on PPIE group’s feedback. PPIE helped us find potential explanations for identifying purpose in life as a key outcome. We discussed what this meant in terms of return to work being a primary outcome. The PPIE clarified that it is possible the return-to-work was an already implied as a key outcome and that stopping work can motivate patients make decisions about what is important in their lives. These suggestions were part of the discussion of the published paper. - PPIE group contributors co-authored this paper. | - Impact - Working together |
| January 2022 | PPIE group provided feedback on findings from the feasibility study to be addressed for the main trial (WP3) and process evaluation (WP4) | - Identified areas to be considered in the main trial: - Discussed how many people returned to less than 80% of preinjury working hours to ensure the right outcomes are measured - Discussed how much intervention is being delivered face-to-face and why, to check if primary delivery method is being maintained - Considered if repatriation to brain injury rehabilitation units excluding potential patients with TBI? Are we losing patients because they do not regain capacity with 12-week inclusion window? - Considered if we should consider removing living within major trauma centre catchment area as an inclusion criterion? This may have therapist capacity implications - Identified that not being able to interview employers meant we were missing an important stakeholder viewpoint - Worked with a PPIE group member with HR experience to improve employer facing materials and develop a new way of approaching employers that doesn’t involve the patient - Got feedback from employer representatives on steering committee to improve employer engagement - Devised employer engagement event as new approach method | - Impact |
| February 2022 & October 2022 | Following feedback from the programme steering committee chairman, discussed adverse events and how we could measure them | - Continued to measure RUSAEs through questionnaires sent to participants and identification from therapists - Continued to discuss potential SAE in mentoring, now with extended list of potential SAEs identified by PPIE group - Discussed with TMG if we need to be measuring SAEs any other way | - Working together |
| May 2022 | Provided extensive feedback on how we can improve follow up rates | - Brought all feedback to recruiting teams on how they can improve their process - Made sure recruiters clearly explaining study and what is expected of participants - Explained importance of returning questionnaires even if haven’t returned to work and if in control group - Highlighted that once questionnaire completed, they will receive their £10 voucher - Made sure participants have phone number of the person completing priming call by sending text message prior to call to identify self. Also make sure recruiters know who is doing priming calls for their site. - Maintain frequent contact with patients (especially those in control group) for duration of trial, so participants know they are still taking part in a trial. Doing this through priming calls and have also developed a patient-facing newsletter to keep them updated on the trial. - Suggested that using phone as primary data collection method may be more powerful. Plan to try this out once ethical approval received on shortened questionnaires - Suggested that follow up questionnaires should be reduced in length (see below) | - Working together - Impact |
| Summer-Autumn 2022 | PPIE group consulted on what should be removed from the questionnaire with aim to improve return rate | - Chief investigator (DK) and health economists (MJ, CR) made suggestions on what could be removed from questionnaires. Then meetings held with PPIE group to discuss what their thoughts on those proposals were. - PPIE group members then completed shortened questionnaires to find out if they were significantly shorter and if there were difficulties completing over the phone - PPIE group members provided more feedback on psychological measures after some members of the PMG expression some concern at their reduction. This resulted in the 2 psychological measures (GAD, PHQ) being kept the same and 1 (IES) being reduced. | - Communications - Impact |
| March  2023 | PPIE group discussed the importance of OTs and how they could best support future patients. Employers should be more involved and aware of patients’ impairments. Discussed OT experiences.  Possible workshop with OTs? Upskilling of OTs mentioned.    A PPIE group member asked why a certain region was not being considered. | - PPIE group’s ideas were turned into some guidance for OTs and PPIE group will be asked to review the guidance - Document was created to support therapists’ discussions with employers to provide confidence in their role and reassurance to employees who don’t want to give consent for employer contact. - Workshop ideas were discussed. - Birmingham was brought to the next TMG for consideration. (It was decided that it was not an appropriate time to introduce a new site so near to the study’s completion) | - Communication - Input |
| July  2023 | PPIE group members give possible solution to patient disengagement.    PPIE group members provide their thoughts and ideas on what will be important to consider in implementation. | - Suggestions from PPIE group included into guidance for therapists. - PPIE group involvement in WPs was discussed in the next meeting. - PPIE members were contacted about patient interviews. - Next meeting was hybrid. | - Working together |
| Dec  2023 | PPIE group suggested that two weeks from injury to recruitment into the study is too short a time.    PPIE group members highlighted poor mental health post injury/ psychological impact of trauma. | - The best time for the intervention to start was considered. - Used meeting notes to create activity log to show the demonstratable impact of PPIE group on study; could use our ‘You Said, We did’ table. - Used questionnaire of PPIE group members and researchers to look at their perspectives on impact. - Created this PPIE input log to show when and how much the PPIE group had been involved. | - Input - Communication |

**Supplementary Table 2: PPIE and researcher questions informed by Mann et al (2018)**

| PPIE questionnaire  Demographic details (Dob, Injury types, date of injury, ethnicity, date began ROWATATE involvement, previous PPI experience)   1. What did you hope to get from being part of ROWTATE PPI group and did you achieve this aim? 2. Why do you think it is important that the PPI group was involved in the ROWTATE study? 3. What do you think has been the most important contribution made by the PPI group and by you? 4. What influence do you think the PPI group has had on the ROWTATE study? 5. What influence do you think being involved in the ROWTATE study has had on you? 6. What has given you the greatest satisfaction in working with the ROWTATE study? 7. What do you think helped you be able to take part in the ROWTATE PPI group? 8. What do you think hindered your ability to take part in the ROWTATE PPI group? 9. Has being part of the ROWTATE PPI group made you more likely to participate in PPI work in the future? Why? 10. Due to the COVID-19 pandemic PPI meetings transitioned from being held in person to online, then afterwards were hybrid (Both online and in person). What do you think the impact of this was? 11. What would you like to be the long term impact of the PPI group? 12. Is there anything else that you would like to add? |
| --- |
| Researcher Questionnaire  Demographic details (DOB, Role on ROWTATE study)   1. Why do you think it is important that the PPI group was involved in the ROWTATE study? 2. What do you see as the PPI group's purpose in the ROWTATE study? 3. What do you think has been the most important contribution made by the PPI group? 4. What influence do you think the PPI group has had on the ROWTATE study? 5. What influence do you think being involved with the PPI group has had on you? 6. What has given you the greatest satisfaction in working with the PPI group? 7. What do you think has helped or hindered the PPI contribution to the ROWTATE study? 8. What (if anything) has been difficult or challenging in working with the PPI group (personally or for the trial)? 9. Due to the COVID-19 pandemic PPI meetings transitioned from being held in person to online, then afterwards were hybrid (Both online and in person). What do you think the impact of this was on the PPI group? 10. What do you like to be the long term impact of the PPI group? 11. Is there anything else you would like to add? |
